# More bang for your buck: potential gains through optimizing maize breeding schemes in sub-Saharan Africa

**DOI:** 10.3389/fpls.2025.1553272

**Published:** 2025-06-03

**Authors:** Davison Chaingeni, Ronica Mukaro, Clay Sneller, Jill E. Cairns, Lennin Musundire, Biswanath Das, Olivia Odiyo, Sammy Madahana, Purity Mazibuko, Washington Mubvereki, Boddupali M. Prasanna, Dumisani Kutywayo

**Affiliations:** ^1^ Crop Breeding Institute, Agricultural Research, Innovation and Specialist Services Directorate, Harare, Zimbabwe; ^2^ Department of Horticulture and Crop Science, The Ohio State University College of Food, Agriculture and Environmental Science, Columbus, OH, United States; ^3^ Global Maize Program, International Maize and Wheat Improvement Centre (CIMMYT), Harare, Zimbabwe; ^4^ Accelerated Breeding Initiative (ABI)-Transform, International Maize and Wheat Improvement Centre (CIMMYT), Nairobi, Kenya; ^5^ Global Maize Program, International Maize and Wheat Improvement Centre (CIMMYT), Nairobi, Kenya

**Keywords:** continuous improvement, deterministic model, genetic gain, breeding efficiency, breeding optimization

## Abstract

Increasing the rate of genetic gain in breeding programs is a critical component of crop genetic improvement strategies to increase yields in smallholder farmers’ fields. While a growing array of technologies and tools are being deployed within breeding programs, optimizing resource allocation could provide a simple yet effective way to increase genetic gain, particularly within resource-constrained breeding programs. The objective of this study was to demonstrate that an easy-to-use deterministic model and a breeding costing tool could identify key modifications to improve the efficiency of breeding within the Zimbabwean national maize breeding program. The current program uses pedigree inbreeding, with a 4–1–1 tester scheme, and relatively low selection intensity. The method of inbreeding, test-crossing schemes, and selection intensity were modified within the current program budget. A combination of using doubled haploid lines, a 2–2–1 tester plan, and increased selection intensity improved gain per cycle by 42.8%, gain per year by 161.8%, gain per dollar by 43.1%, and decreased cost of one unit of genetic gain by 28.5% without a change in budget. Our results highlight how a simple deterministic model can identify steps to greatly improve breeding efficiency within resource-constrained breeding programs.

## Introduction

Demand for maize in sub-Saharan Africa (SSA) is expected to increase 2.3-fold over the next 30 years (Aramburu-Merlos et al., 2024; van Ittersum et al., 2016). The estimated gap between farm yield and potential yield in SSA is 80% (Giller et al., 2021), which is increasing over a large proportion of the region (Gerber et al., 2024). Multiple interventions will be required to sustainably reduce maize yield gaps in this region (Cairns et al., 2021; Jain et al., 2023; Aramburu-Merlos et al., 2024). Improved genetics have historically played a key component in strategies to increase crop yields and productivity (Pingali, 2012; Hansen et al., 2019). The ability of improved genetics to reduce the yield gap in farmers’ fields is dependent on population improvement, genetic gain for key traits, and the rate of varietal replacement (Atlin et al., 2017). Over the past two decades, significant investment has been made in strengthening the maize seed value chain within SSA (Chivasa et al., 2022). The area planted with new stress-tolerant hybrids across eight countries in eastern and southern Africa (ESA) is estimated to have increased over threefold to almost 5 million hectares between 2016 and 2021 (Cairns and Prasanna, 2018; Chivasa et al., 2022). The maize yield gap is a function of many factors, particularly agronomy. Although greater access to improved genetics (alongside other interventions) has not resulted in yield increases at the national level in many countries, except for Ethiopia (Chivasa et al., 2022), there is growing ex-post evidence of the impact of improved maize varieties on farm-level production, household income, and reducing the depth of poverty (Lunduka et al., 2019; Katengeza and Holden, 2019; Martey et al., 2020; Gebre et al., 2021; Habte et al., 2023; Ngoma et al., 2025). The recent emphasis on improving the efficiency of maize breeding programs is translating to increased genetic gain in grain yield by both public and private breeding programs in SSA (Masuka et al., 2017a, b; Kebede et al., 2020; Prasanna et al., 2022; Asea et al., 2023; Ligeyo et al., 2024; Mazibuko et al., 2024; Mukaro et al., 2024). Despite progress, higher rates of gain are required to reach the estimated annual yield gain of 2.4% required to meet future demands (Ray et al., 2012).

There is a vast amount of literature proposing new methodologies, tools, and technologies to increase genetic gain within crop breeding programs (Shakoor et al., 2017; Li et al., 2018; Xu et al., 2018; Yang et al., 2020; Sandhu et al., 2021; Farooq et al., 2024). Proposed technologies include the use of proximal and remote sensing tools to increase the accuracy of field phenotyping (Araus et al., 2018) and phenotyping new traits for inclusion in advancement decisions (Yang et al., 2020), rapid generation advancement methods to reduce the time taken for homozygous line development (Lenaerts et al., 2018a; Watson et al., 2018; Lenaerts et al., 2021), and genomic prediction to select individuals or families prior to field phenotyping (Choquette et al., 2023). Bernardo (2021) proposes three technologies and resources routinely used in commercial maize breeding: two cycle genome-wide selection, double haploids, and continuous nurseries to enhance genetic gain in a breeding program. Although it is difficult to partition changes in genetic gain over time to the adoption of individual methodologies and tools within a breeding pipeline, there is a growing body of evidence linking the adoption of these tools and technologies by breeding programs to higher rates of gain (Cooper et al., 2014; Yadav et al., 2021; Prasanna et al., 2022). Cooper et al. (2014) highlighted expanding phenotyping networks as a key component of increased genetic gain in USA maize breeding. Recent gains in the USA corn belt have been attributed to using genomic prediction technologies to select individuals or families prior to field testing (Messina et al., 2020). Across six breeding pipelines in ESA, Prasanna et al. (2022) found the highest gains were made in the breeding pipeline that was the first to adopt DH production for inbred line development, forward breeding for key diseases, and genomic selection for grain yield under drought and optimum conditions.

**Figure 1:**
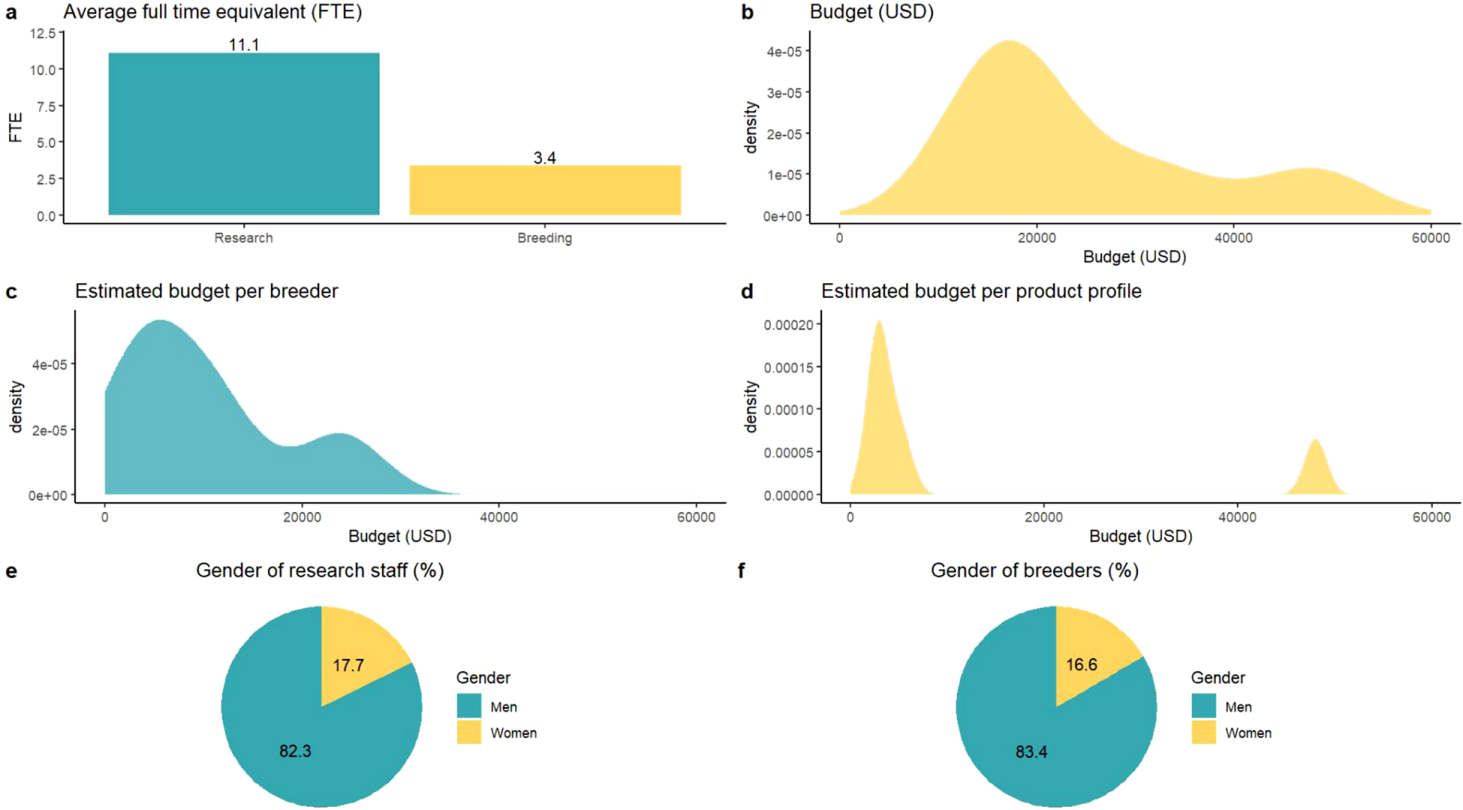
Overview of survey conducted by the Consultative Group on International Agricultural Research (CGIAR) Excellence in Breeding Platform with seven national maize breeding programs in eastern and southern Africa with **(a)** average FTE of researchers and breeders, density plots of **(b)** operational budget across maize breeding programs, **(c)** estimated operational budget per breeder, **(d)** estimated budget per product profile, and gender of **(e)** research staff and **(f)** breeders.

The ability to deploy new tools, technologies, and methodologies to increase genetic gain is partially a function of budget and capacity. Budget and capacity are often major limitations for public sector breeding programs (Cobb et al., 2019; Coe et al., 2020; Egan et al., 2024). A public-sector plant breeding program survey in the USA found an average of 1.58 full-time equivalent (FTE) devoted to germplasm enhancement and 2.2 FTE dedicated to variety development (Coe et al., 2020). The average operating cost of US public sector breeding programs was 266,562 USD, although in the US, maize improvement has been driven by the private sector (Renkow, 2019). The average number of national or Consultative Group of International Agricultural Research (CGIAR) plant breeders per country across 30 African countries was estimated at five (Walker et al., 2014; Suza et al., 2016). A recent CGIAR Excellence in Breeding Platform survey of breeding capacity in seven countries in ESA found that national maize breeding programs had an average of 11.1 FTE for research staff and an average FTE of 3.4 for breeders (**Figure 1a**) (Excellence in Breeding (EiB) program). The estimated operating budget was approximately 25,000 USD (**Figure 1b**), equating to an average operating budget of 10,044 USD per breeder (**Figures 1c, d**). This cost is lower than the estimated 16,800 USD for one bi-parental population in the private sector in the US (Bernardo, 2021). This cost assumed developing 150 doubled haploids, testcrossing doubled haploids to one tester, and phenotyping testcrosses at six locations. Among seven NARES breeding programs in ESA, the average FTE for biometrician support was 0.11, with two national maize programs lacking statistical support. The number of product profiles targeted by each program ranged from one to seven. Interestingly, <20% of researchers or breeders were women (**Figures 1e, f**), confirming the continued gender (sex) disparity in breeding programs within SSA (Diop et al., 2013; Pixley et al., 2023; Rice et al., 2024).

Given the budget and personnel constraints, many public sector breeding programs face challenges to actively deploy proven tools and methodologies to increase genetic gain. While several studies document the benefits of new tools and technologies to influence parameters of the “breeders equation” towards increased genetic gain (Araus et al., 2018; Dieng et al., 2024), few studies articulate requirements (including financial) to be able to deploy innovative solution and highlight potential limitations that may restrict adoption by resource-constrained breeding programs. For example, speed breeding manipulates the photoperiod within a day to increase the rate of development of plants and ultimately reduces generation time (Jähne et al., 2020). Up to six generations of spring wheat, barley, and chickpea can be achieved within a year using speed breeding (Watson et al., 2018). However, deploying speed breeding within a breeding program requires moderate to high initial capital investment (Wanga et al., 2021). Furthermore, this approach has large energy requirements, with temperature regulation estimated to account for over half of the total plant management costs (O’Connor et al., 2013). Access to a regular, stable electricity supply is difficult in many countries. High throughput phenotyping platforms and tools can provide more precise and accurate estimates of the genetic value of individuals (Araus et al., 2018). Remote and proximal sensing platforms can also reduce the cost of labor to acquire measurements and increase selection accuracy, particularly when used as an alternative to visual scores of senescence. However, they require initial investment in capital and technical capacity for data processing (Pauli et al., 2016). As mentioned, analytical capacity is limited in many public sector breeding programs. In self-pollinating crops where manipulating growth conditions within a greenhouse or screenhouse can induce earlier flowering and seed set, rapid generation advancement (RGA) or single seed descent (SSD) can fix lines rapidly. There remains a low adoption of RGA within public sector rice breeding programs despite its proven benefits due to constraints faced by these programs. Collard et al. (2017a, b) estimated initial investment and operational costs for RGA relative to the pedigree method for inbred line development. Compared to the pedigree method, RGA costs approximately one-seventh of the cost to generate 10,000 breeding lines. While initial investment costs are high with RGA, costs associated with infrastructure development are repaid within the first year of using RGA. Decreasing genotyping costs have increased the accessibility of molecular-based strategies. Genomic selection for grain yield under well-watered and drought stress reduced the cost of hybrid maize development by an estimated 32%, associated with a reduction in field phenotyping requirements (Beyene et al., 2019). However, significant quantitative genetics support is required to routinely deploy genomic prediction in selecting individuals prior to field phenotyping. Furthermore, the costs associated with building the initial training set to make accurate predictions for quantitative traits such as grain yield are high. Thus, while there is an ever-increasing array of new methodologies, tools, and technologies available to increase the rate of genetic gain within breeding programs, practical considerations (including budget constraints) may limit their uptake.

Acknowledging both the constrained capacity of national breeding programs in the Global South and the need to increase gains, optimizing current “traditional” breeding schemes could provide an important pathway towards increasing breeding efficiency and, ultimately, the rate of genetic gain for key traits (Barros et al., 2018). Breeding schemes can be defined as “a collection of crossing, evaluation, and selection tasks and decisions which vary across breeding stages (e.g., in the crossing block, advanced yield testing in plants) and ultimately define a breeding strategy” (Covarrubias-Pazaran et al., 2022). There are various simulation models to compare the efficiency of different breeding schemes (Sun et al., 2011; Faux et al., 2016; Bernardo, 2017; Villiers et al., 2022). However, the ability to apply simulation modeling requires sufficient technical capacity. In addition, many models do not incorporate operational costs within their simulations (Faux et al., 2016), assume a fixed cost per row across locations (Bernardo, 2021), or use estimated plot units (Schoemaker et al., 2024). In breeding programs serving highly competitive markets and in which advanced breeding tools and technologies have already been deployed, simulation models and prediction tools can play an important role in resource allocation decisions to increase the rate genetic gain (Bernardo, 2021; Peixoto et al., 2024; Schoemaker et al., 2024). However, these studies have generally been conducted within programs where breeding schemes were optimized prior to the application of advanced breeding tools and technologies, with budgets significantly higher than that of public sector breeding programs serving crop improvement in SSA. Given that resources are a major constraint within national breeding programs, it is important to understand the impact of potential changes within the context of their own realities. Atlin and Econopouly (2022) developed a simple deterministic model to compare predicted selection responses per cycle across different breeding schemes and resource allocation. Cost considerations are important in terms of understanding the impact of potential changes in breeding schemes within the context of a program’s current budget. This allows breeding programs to assess the economic viability of different breeding strategies and investments. The model incorporates operational costs to facilitate quantifying potential gains in using different breeding schemes within a fixed budget through optimized resource allocation. The aim of this study was to quantify potential gains that could be made within a fixed budget through improved resource allocation. The Breeding Costing Tool (University of Queensland, 2018) was used to quantify breeding costs, which were integrated into the deterministic model (Atlin and Econopouly, 2022) to compare the genetic gain and breeding efficiency metrics of different breeding schemes quantitatively. For this study, we focused on the maize breeding program of the Agricultural Research, Innovation, and Specialist Services (ARISS) Directorate of the Government of Zimbabwe as an example of a national maize breeding program focused on continuous improvement, which occupies approximately 15% of the maize seed market in Zimbabwe (Mukaro et al., 2024). ARISS program accounts for approximately 90% of the Pro-vitamin A hybrids on the market in Zimbabwe.

## Methods

### Product profiles, breeding pipelines, and schemes

**Figure 2:**
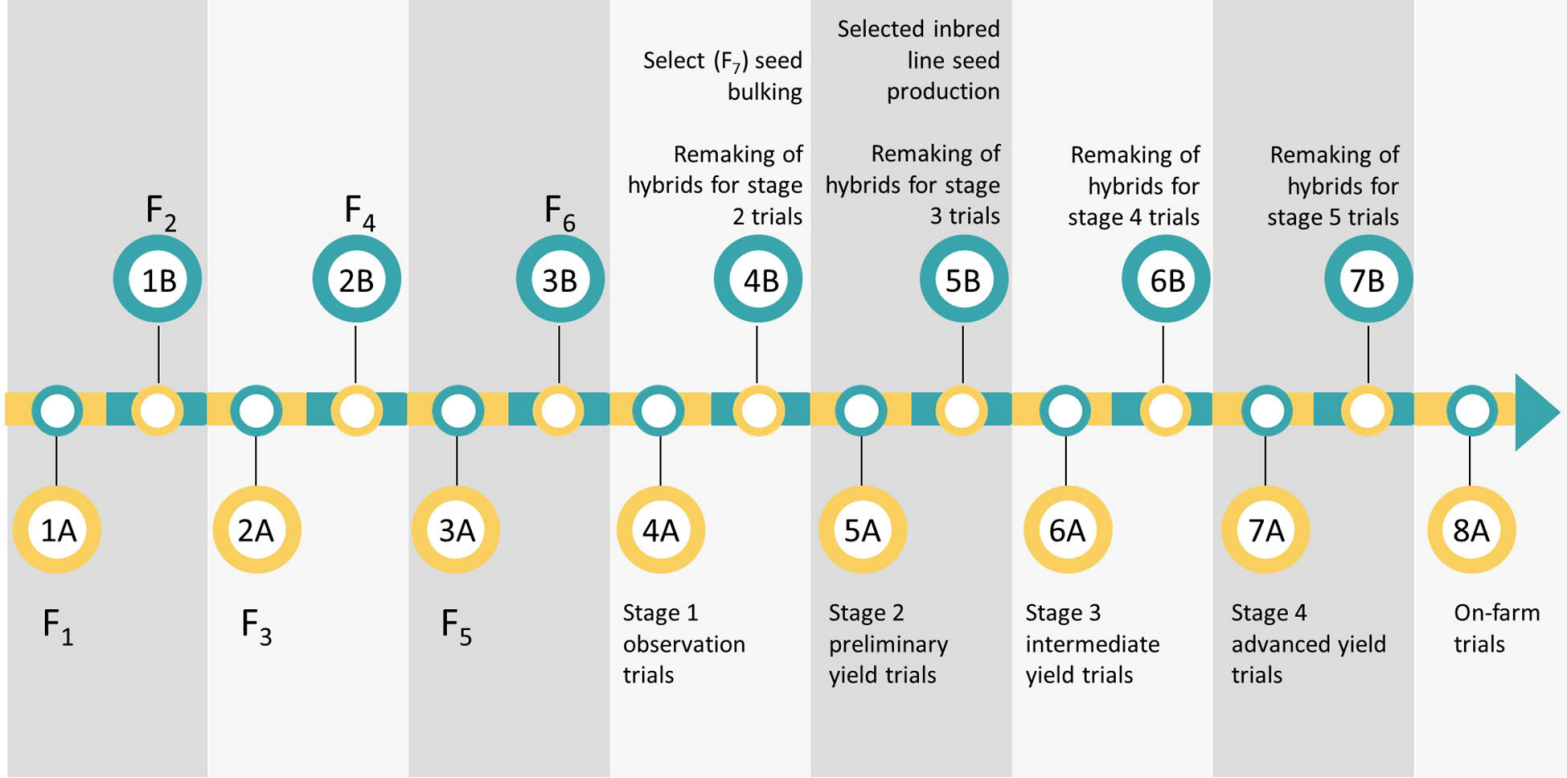
Overview of the maize breeding scheme currently used by the Agricultural Research, Innovation and Specialist Services (ARISS) Directorate of the Government of Zimbabwe. Pedigree breeding is used for inbred line development to F6. Testcrosses are evaluated in observation trials, preliminary yield trials, intermediate yield trials and finally, advanced yield trials. The most promising candidate hybrids are moved to on-farm trials for farmer evaluation prior to commercialization. Each grey panel graphically represents six-months.

**Figure 3:**
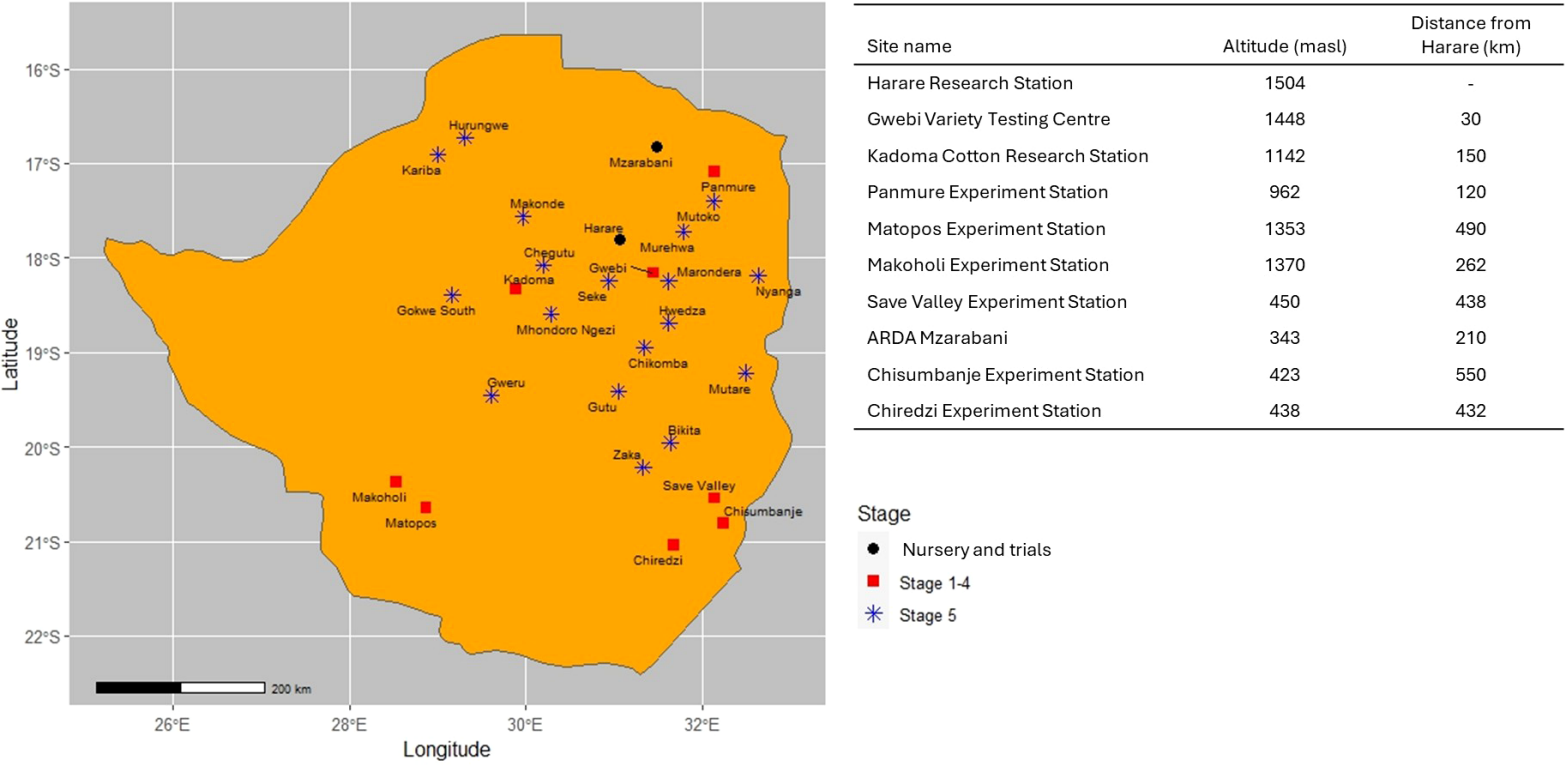
Overview of nursery and testing locations and distance to Harare used by the Agricultural Research, Innovation and Specialist Services Directorate of the Government of Zimbabwe national maize breeding program.

The ARISS maize breeding program targets four product profiles: ultra-early to very early maturity, early to medium maturity, late maturity, and biofortification (provitamin A) maize. Natural regions, must-have traits, and market share of each breeding pipeline are presented in **Table 1**. The current breeding schemes are similar across the four breeding pipelines (**Figure 2**). Briefly, bi-parental crosses are made, and pedigree breeding is used to advance population to the F_6_ generation using two nurseries per year. Four testers are used to test cross inbred lines and assign them to different heterotic groups upon the development of homogenous inbred lines. They also establish the inbred lines’ general combing ability and specific combining ability. The number of inbred lines and test crosses varies by breeding pipeline (**Table 2**). Inbred lines are advanced based on their testcross performance across multiple locations (Stage 1 to Stage 4) until on-farm trials (Stage 5) (**Figure 3**). Lines with superior performance are selected from the Stage 3 trials and are used as parents to initiate a new breeding cycle. When advancing through the testing stages, selection intensity ranges from 17% to 80% (**Table 2**). Genetic trends for grain yield over the past 20 years within the ARISS maize breeding program were recently estimated at 16 kg ha^−1^ year^−1^ in low-yield potential environments and 61 kg ha^−1^ year^−1^ in high-yield potential environments (Mukaro et al., 2024).

**Table 1:** Summary of the Directorate of Agricultural Research and Specialist Services (ARISS) of the Government of Zimbabwe, breeding pipelines, including natural regions, each pipeline targets, traits and market share of each breeding pipeline.

Product profile	Market share (%)	Maturity range	Natural region	Traits required for advancement							
				Grain yield under		Maize streak virus	Gray leaf spot	Turcicum leaf blight	Husk cover	Lodging	Pro-vitamin A content
				Drought and heat	Low N						
Ultra-early to very early maturity	35	90-120	III-V		x	x	x	x	x	x	
Early to medium maturity	45	120-149	II-IV	x	x	x	x	x	x	x	
Late maturity	15	≥150	I & II	x	x	x	x	x	x	x	
Nutritious	5	≥150	I & II	x	x	x	x	x	x	x	x

Available online at: https://www.cimmyt.org/b;ogs/product-design-teams-pdts-aclient-oriented-approach-to-defining-market-segments-and-target-productprofiles/.

**Table 2:** Summary of the average number of lines at each stage of inbred line development and the number of lines at each trialling stage of the breeding pipeline for the Directorate of Agricultural Research and Specialist Services (ARISS).

Stage	Ultra-early to early	Early to intermediate	Late	Nutritious
F_1_	18	38	18	10
F_2_	18	38	18	10
F_3_	300	600	200	150
F_4_	150	300	100	75
F_5_	60	100	40	30
F_6_	25	50	20	15
F_7_	25	50	20	15
Stage 1	75 (80%)	200 (43%)	50 (90%)	75 (47%)
Stage 2	60 (67%)	85 (71%)	45 (89%)	35 (57%)
Stage 3	40 (20%)	60 (25%)	40 (20%)	20 (25%)

Selection intensity is included in parentheses.

### Costing breeding pipelines

The deterministic model required the following costs: 1) cost of making a cross and advancing to the F_2_ generation, 2) cost to develop an inbred progeny, 3) cost to make a testcross with enough seed for testing, 4) cost to phenotype a Stage 1 plot, 5) cost to phenotype a Stage 2 and Stage 3 plot, 6) cost to assess pest resistance, 7) cost to assess quality, and 8) costs of personnel assigned to the breeding program. The costs of (4) and (5) were allowed to vary by whether the trial was conducted on- or off-station.

The cost of each breeding pipeline component was obtained using the Breeding Costing Tool version 1.16.7.0 (University of Queensland, 2018). The Breeding Costing Tool calculates the cost of running a breeding pipeline using a modular framework incorporating unit costs (item, supplies, operational and fixed costs, and a cost for facilities and administration) for each breeding operation (**Table 2**) and the cost of defined activities to within each breeding pipeline. The Breeding Costing Tool determined the cost of a 5-m row; thus, the actual costs of a row in each stage of the ARISS pipeline were adjusted based on the row length used. The costs per 5-m row varied by pipeline and across advancement stages. The ARISS maize breeding program currently uses a modified pedigree and partially backcross breeding methods during generation advancement using two seasons in a year between the main season (Harare) and the off/winter season in Muzarabani (**Table 3**), and selections are planted ear-to-row between generations. Muzarabani is located 210 km from Harare, where ARISS is located.

**Table 3:** The relative cost of a yield plot by pipeline, testing stage, and location expressed as the percentage of the cost of a locally managed plot in the early-intermediate maturity program, is shown in underlined italics.

Stage	Location	Early to intermediate	Ultra-Early	Late	Nutritious
Stage 1	Local	*100*	158	173	145
Stage 2	Local	*100*	121	115	118
	Distant	107	163	163	160
Stage 3	Local	*100*	137	137	138
	Distant	136	220	220	240

The Agricultural Research, Innovation, and Specialist Services (ARISS) Directorate headquarters are in Harare and trials within Harare are referred to as local and those outwith as distant.

### Cost of inbred line and testcross production

Current breeding pipelines use a modified pedigree breeding scheme to the F_6_ generation. The first required cost is generating F_2_ seed (**Table 4**). The program uses one 4-m row per parent and one row for each set of F_1_ per cross in all four breeding pipelines. When the F_1_ is created, leaf samples are submitted for genotypic quality control to confirm that the F_1_ is derived from the specific parents and to rule out potential cross-contamination (Gowda, 2017). The cost per 4-m row varied considerably across breeding pipelines. The cost of generating F_4_, F_5_, or F_6_ inbred lines from an F_2_ population using either the pedigree or SSD systems was estimated for each breeding pipeline (**Table 4**). The costs of growing all 4-m rows in the F_2_ to F_n_ nurseries were added and divided by the number of inbred lines placed in Stage 1 testing to estimate the cost of one inbred line. The Breeding Costing Tool was used to determine the cost of the pedigree breeding scheme.

**Table 4:** Costs associated with generating F2 seed with the pedigree or single seed descent (SSD) system in the four Agricultural Research Innovation, Specialist Services (ARISS) Directorate maize breeding pipelines.

Pipeline	Activity	Cost as % of the cost of the early-inter maturity pedigree scheme			
		Cost of the pedigree scheme		Cost of SSD scheme	
		Cost of activity	Total cost	Cost of activity	Total cost
Ultra-Early	Plant parents	100	137	100	68
	Generate F_1_ seed	168		42	
Early-inter.	Plant parents	*100*	*100*	100	59
	Generate F_1_ seed	*100*		25	
Late	Plant parents	100	143	100	70
	Generate F_1_ seed	178		45	
Provitamin A	Plant parents	100	195	100	83
	Generate F_1_ seed	273		68	

Costs are expressed as a percentage of the costs of generating lines in the intermediate maturity pipeline, shown in underlined italics.

The cost of developing inbred lines via SSD was estimated by working backwards from the target number of Stage 1 lines (**Table 2**). We assumed a 30% loss of plants between generations due to failure to germinate or selection. For example, to generate 300 F_6_-derived lines, 390 F_5_ plants, 507 F_4_ plants, 660 F_3_ plants, and 858 F_2_ plants needed to be generated. We assumed a planting density of four seeds per meter. The length of row required to grow that number of plants and the cost per meter of row were used to calculate the cost of the SSD method. The SSD system requires fewer F_1_ plants, so the row length of the F_1_ nursery was set at 2.5 m versus 4 m for the pedigree scheme. The cost of creating a DH line is presented in **Table 5**. The DH scheme also requires fewer F_1_ plants and was costed assuming 2.5 m row length. We assumed a DH line was selfed for one generation prior to being used as a parent to generate Stage 1 testcrosses, and the seed of the selfed plant were grown using ear-to-row selections in 4-m row plots. The final pre-advancement cost was the cost of forming testcrosses, and this cost was obtained directly from the Breeding Costing Tool.

**Table 5:** Costs associated with generating F_4_, F_5_, F_6_ and double haploid derived lines for each of the four Agricultural Research Innovation, Specialist Services (ARISS) Directorate maize breeding pipelines.

	Generation of lines	Cost as % of early-intermediate F_6_-derived line		
		Pedigree	SSD	DH
Early to intermediate	F_6_	*100.0*	12.3	
	F_5_	96.7	8.4	
	F_4_	87.0	5.4	
	DH			62.8
Ultra early	F_6_	174.5	14.1	
	F_5_	164.1	9.7	
	F_4_	131.1	6.2	
	DH			60.4
Late	F_6_	164.6	18.2	
	F_5_	147.5	12.4	
	F_4_	133.5	8.0	
	DH			60.4
Nutritious	F_6_	95.1	20.5	
	F_5_	80.4	14.0	
	F_4_	72.5	9.0	
	DH			62.8

Costs are expressed as a percentage of generating an F_6_ lines by the pedigree method in the intermediate maturity breeding pipeline, shown in underlined italics.

### Cost of product advancement stages

The cost of conducting a trial was determined by calculating the cost per plot for each stage of advancement multiplied by the number of plots. The cost per plot was determined by the number of rows per plot and row length. The costs per plot varied by program, advancement stage, and the location where trials were conducted (**Table 3**). The Breeding Costing Tool estimated costs for each location, including travel costs, and these costs were averaged to calculate a common plot cost for all locations outside Harare, including all associated travel costs. The deterministic model also required fixed costs of managing a location, costs for disease resistance screening, fixed costs for staff time, facilities, and administration charges. These costs were assumed to be the same for all breeding pipelines and evaluated schemes.

### Modelling breeding schemes

To model the effect of different inbred line development schemes, the number of testcrosses used, and selection intensity on genetic gain Equation 1, an Excel-based simple deterministic simulation model developed by Atlin and Econopouly (2022) was used. This model required the cost estimates described above and estimates of heritability (Equation 2) and genetic variances. The model utilizes the breeder’s equation and estimates the response to selection of one trait as:


(1) $$G_{i}=k_{i}k\sigma_{a}{\sqrt{H}}_{i}$$

where G_i_ is the genetic gain from the *^i^* ^th^ stage, k_i_ is the standardized selection differential when advancing lines from the *i* ^th^ stage to the *i* ^th^+1 stage, $\sigma_{a}$ is the standard deviation of the additive genetic variance, and √H_i_ is the square root of the entry mean heritability within the *i* ^th^ stage.

Grain yield data from Stage 2 and 3 trials of the ARISS early to intermediate maturity pipeline was previously analyzed by Mukaro et al. (2024). They provided genetic, genotype × environment estimates, and error variance to estimate heritability. The average of the variance components across the two stages of trials were as follows: genotype variance = 263,256 (11.5% of the total variance), genotype by environment variance = 306,396 (13.5% of the total variance), and error variance = 1,711,060 (75% of the total variance). Entry mean heritability (EMH) was calculated within each stage of testing as


(2) $$H=\frac{\sigma_{g}^{2}}{\sigma_{g}^{2}+\langle \frac{\sigma_{gl}^{2}}{l}\rangle +\langle \frac{\sigma_{e}^{2}}{lr}\rangle }$$

where $\sigma_{g}^{2}$ is genetic variance, $\sigma_{gl}^{2}$ is the genotype by location variance, $\sigma_{e}^{2}$ is the error variance, *l* is the number of test environments within a testing stage, and r is the number of replications.

Only breeding schemes where the parents were selected after Stage 3 were modeled. We assumed five or seven inbred lines were selected from Stage 3 to be used as parents to initiate a new cycle, and these numbers were used to calculate Stage 3 selection intensity. The standard deviation of the genetic variance among lines was used as a surrogate for $\sigma_{a}$ . The model adjusts genetic variance for the degree of inbreeding of the lines. Total genetic gain per cycle (G_c_) was estimated as the sum of genetic gain from each stage of testing.


(3) $$G_{c}=\sum_{i=1}^{3}G_{i}$$

The efficiency and effectiveness of the different breeding schemes were estimated as 1) gain per cycle (G_c_), 2) gain per year (G_y_ = G_c_/years per scheme), 3) total cost of a cycle ($_t_), 4) gain per USD (G_c_/$_t_), and 5) cost of one unit of genetic gain ($_g_=$_t_/G_c_).

### Modifications to the deterministic model

Several changes were made to the Atlin and Econopouly (2022) Excel file and model. In the original Excel file, the cost per plot was assumed to be constant across all testing locations and stages of testing. The modification allowed costs to vary by location and testing stage, as the number of rows per plot and row length varied. The model was adapted to incorporate the cost of disease screening at every stage of the breeding pipeline. The spreadsheet was modified to allow the number of testers to vary between stages. The original model calculated the annual cost of conducting the entire program, assuming crossing, inbreeding, and all testing stages occurred yearly. While this is likely, we modified the model to calculate the cost per cycle so that the cost of each operation was only used once to calculate the cost of one breeding cycle.

Several components of breeding schemes were modified from the current scheme within each of the four pipelines, and their impact on efficiency parameters were compared. Efficiency parameters were calculated for each scheme and then expressed as a percentage of the current scheme. The current scheme of each pipeline was inbreeding to the F_6_ generation using pedigree breeding. New parents were selected after the third stage of testing. The first comparison was generating inbred lines via pedigree, SSD, or DH. We compared inbreeding using pedigree or SSD to the F_4_, F_5_, or F_6_ generation. We then compared two tester schemes. The current scheme uses four testers in Stage 1 and one tester per inbred line in Stages 3 and 4 (referred to as the 4–1–1 scheme). We assessed an alternative breeding scheme using two testers in Stage 1, two testers in Stage 2, and one tester per inbred line in Stage 3 (referred to as the 2–2–1 scheme). We compared schemes that varied in selection intensity. However, it should be noted that this is not the best industry practice, as advanced breeding programs use one tester on each side of heterotic grouping (A and B) and more testers in advanced testing stages. We first compared schemes, allowing total costs per cycle (S_t_) to vary by scheme. We then compared the modified schemes to the base scheme by constraining the total cost of all breeding schemes to equal the cost of the base pipeline ($_b_). If the pipeline modifications cost less than the base pipeline, the savings were applied to increase the number of lines in the early testing stages until the new scheme’s total cost was the same as the current (base) scheme. All costs and parameters of genetic gain and efficiency were expressed relative to those of the current (base) pipeline for each of the four product profiles.

## Results

Changes in breeding schemes were modeled across all four pipelines. Each pipeline employed the same base breeding scheme of pedigree breeding to the F_6_ generation, crossing each Stage 1 line to four testers and crossing each Stage 2 and 3 line to one tester. After Stage 3, eight lines were selected for crossing at the end of a cycle for the large intermediate-maturity breeding pipeline, and five lines were selected for crossing at the end of the cycle for the other three smaller pipelines (**Table 2**). The impact of the modifications was similar within each of the four pipelines; therefore, the average across all pipelines is presented.

### Comparison of inbred line development schemes

#### Variable budget across inbred line development schemes

The pedigree scheme with inbreeding to the F_6_ generation (the base scheme) was the most expensive method of inbred line development (**Tables 4**, **5**). The SSD method required fewer and shorter rows than the pedigree method and thus cost considerably less per line. However, it should be noted that for the inbred line development schemes (pedigree, DH, and SSD), there was additional time and cost involved in increasing the seed of the homozygous inbred lines for use in the test crossing, and this was assumed to be fixed cost for all the breeding schemes. Considering costs from parents to F_6_, and averaged across the four pipelines, it costs 83.7% less to generate an F_6_ line by SSD than by pedigree breeding and 38.4% less for a DH line versus an F_6_ pedigree line (**Table 5**). Developing an F_6_-derived line via SSD costs 73.6% less than DH technology. There was little difference in the gain per cycle with less inbreeding (F_4_ versus F_6_), whether using the pedigree or SSD method (**Table 6**). As expected, less inbreeding decreased gain per cycle in the pedigree and SSD systems due to a reduction of genetic variance but improved gain per year (**Table 6**). Less inbreeding decreased gain per USD and increased the cost of one unit of genetic gain in the pedigree and SSD schemes. The DH system resulted in the greatest gain per cycle, gain per year, gain per dollar, and cost of one unit of genetic gain (**Table 6**) when the total cost of a breeding cycle was allowed to vary by scheme.

**Table 6:** Predicted impact of different methods to produce inbred lines on the total cost, genetic gain per breeding cycle, genetic gain per year, gain per USD, and USD per one unit of genetic gain.

Cost Control	Method	Years per cycle	Total cost per cycle	Total gain per cycle	Gain per year	Gain per USD	USD per 1 unit of gain
Vary	Pedigree (Base), F_6_	11	*100.0*	*100.0*	*100.0*	*100.0*	*100.0*
	Pedigree, F_5_	10	99.1	97.4	107.2	98.4	101.7
	Pedigree, F_4_	9	97.7	92.3	112.8	94.4	105.9
	SSD, F_6_	11	88.3	100.0	100.0	113.2	88.3
	SSD, F_5_	10	87.9	97.4	107.2	110.9	90.2
	SSD, F_4_	9	87.6	92.3	112.8	105.4	94.9
	Doubled haploid	6	92.3	102.5	188.0	111.1	90.1
Fixed cost	Pedigree, F_5_	10	99.8	97.8	107.6	98.1	102.0
	Pedigree, F_4_	9	99.7	93.2	113.9	93.5	107.0
	SSD, F_6_	11	99.8	105.5	105.5	105.7	94.6
	SSD, F5	10	99.7	103.0	113.2	103.2	96.9
	SSD, F_4_	9	99.9	97.7	119.4	97.8	102.3
	Doubled haploid	6	99.5	106.1	194.5	106.7	93.7

Values are averaged across the four Agricultural Research, Innovation, and Specialist Services (ARISS) Directorate maize breeding pipelines and presented relative to the metrics of the current base programs which uses pedigree breeding to F_6_ with an 11-year breeding cycle, shown in underlined italics.

#### Fixed budget across inbred line development schemes

The total cost of a breeding cycle (line development plus product advancement) using SSD and DH was approximately 14% and 8% less, respectively, than the cost of the base pedigree inbreeding scheme (**Table 6**). The reduced costs associated with using SSD or DH allowed the reallocation of resources to test more lines in Stage 1. The breeding schemes were subsequently modified to test a higher number of lines in Stage 1 until the new budget of each equaled that of the base scheme within each profile. In the base schemes, an average of 56% of Stage 1 lines were advanced to Stage 2 (**Table 2**). This provided a modest improvement in selection intensity, as the amount of funds re-allocated to increase the size of the Stage 1 trials was not large. The percentage of Stage 1 lines advanced to Stage 2 in the modified schemes was 50% in the SSD (F_6_) scheme and 56% in the DH scheme. The increased Stage 1 selection intensity increased gain per cycle and genetic gain per year but reduced gain per dollar and cost of one unit of genetic gain (**Table 6**). The DH scheme with increased selection intensity improved gain per cycle by 6.1%, genetic gain per year by 94.5%, gain per dollar by 6.7%, and cost of one unit of genetic gain by 6.4% compared to the base scheme (**Table 6**).

### Comparison of breeding efficiency using different number of testers within a fixed budget across schemes

The current breeding schemes crosses each Stage 1 inbred line to four testers due in part to the ambiguous nature of the heterotic groups in southern Africa. This breeding scheme significantly reduces the number of unique genotypes assessed in Stage 1 as each inbred line creates four entities to be tested. Inbred lines advanced to Stage 2 and 3 are each crossed to one tester. This base tester scheme is referred to as the 4–1–1 scheme (four testers in Stage 1, one tester in Stage 2 and Stage 3) and is used in all four pipelines. The impact of implementing a 2–2–1 scheme (two testers in Stage 1, one tester in Stage 2 and 3) was investigated. This scheme enabled the evaluation of more lines in Stage 1 and facilitated a higher selection intensity.

The 2–2–1 scheme reduced the total cost of a scheme by an average of 22.9%, as fewer testcrosses were formed for stage 1 trials. The cost savings were reallocated to increase the size of the Stage 1 trial until the cost of all modified pipelines was the same as the base scheme. The base schemes using the 4–1–1 plan advanced an average of 65% of Stage 1 lines to Stage 2. Under the 2–2–1 modification, with the increased size of the stage 1 trial, the percentage of the line advanced to Stage 1 was 26% for the pedigree scheme, 18% for the SSD scheme, and 21% for the DH scheme. The 2–2–1 scheme increased total gain per cycle by an average of 10.1%, gain per year by 38.7%, gain per dollar by 8.2%, and reduced cost of one unit of genetic gain by 7.4% compared to the base program (**Table 7**). The DH scheme, coupled with the 2–2–1 tester scheme and the resulting increased selection intensity, produced 15.6% greater gain per cycle, 112% greater genetic gain per year, 13.2% better gain per dollar, and 11.9% better cost of one unit of genetic gain as compared to the base scheme. The SSD(F_6_) scheme produced similar gain per cycle, gain per dollar, and $ per 1 unit of gain values as the DH method.

**Table 7:** Effect of utilizing the 2–2–1 tester scheme on breeding metrics expressed as a percentage of the metrics from the base (current) breeding scheme that uses the pedigree method and a 4–1–1– tester scheme.

Inbreeding method and generation	Gain per cycle	Gain per year	Gain per dollar	Cost of one unit of gain
Pedigree, F_6_	109.9	109.9	108.2	92.4
Pedigree, F_4_	101.6	124.2	99.9	100.1
SSD, F_6_	116.2	116.2	114.3	87.5
SSD, F_4_	107.2	131.0	105.1	95.1
Doubled haploid	115.6	212.0	113.5	88.1
Average	110.1	138.7	108.2	92.6

Results are averaged over the four Agricultural Research, Innovation, and Specialist Services (ARISS) Directorate maize breeding pipelines. The total cost of the modified schemes were constrained to equal the total cost of the base scheme.

### Combining modifications of inbreeding, number of testers, and selection intensity within a fixed budget across all schemes

Previous modifications reallocated funds to increase the size and selection intensity in Stage 1. We extended the impact of increased selection intensity to Stage 2 and Stage 3 advancements. This modified selection intensity plan was coupled with the 2–2–1 tester scheme and different inbreeding schemes (**Table 8**). All budgets were constrained to equal the base budget.

**Table 8:** Summary of the average number of lines in each stage of testing and selection intensity for each of the four Agricultural Research Innovation, Specialist Services (ARISS) Directorate maize breeding pipelines.

Status	Breeding pipeline	Average Number of Lines				% lines selected		
		Stage-1	Stage-2	Stage-3	Selected as Parents	1->2	2->3	3-> parents
Base	Early to intermediate	200	85	60	15	43	71	25
Base	Ultra early	75	60	40	8	80	67	20
Base	Late	50	45	40	8	90	89	20
Base	Nutritious	75	35	20	5	47	57	25
Modified	Early to intermediate	249	56	14	7	23	25	50
Modified	Ultra early	109	40	10	5	37	25	50
Modified	Late	78	25	10	5	32	30	50
Modified	Nutritious	88	35	10	5	40	29	50

Values are averaged over all inbreeding methods. Values are shown for the base scheme and for the modifications. The average selection intensity is between Stages 1 and 2, 2 and 3, and 3 and parents.

The base schemes advanced an average of 84% of lines from Stage 2 to Stage 3, and 19.5% of Stage 3 lines were advanced as parents (**Table 8**). We changed the size of Stage 2 and Stage3 trials and selected an average of 27.3% of Stage 2 lines to enter Stage 3, and 50% of Stage 3 lines were advanced to the crossing block (**Table 8**). These percentages varied by pipeline due to the varying size and budgets of the pipelines. Selection intensity from Stage 3 was high as the number of entries in Stage 3 was small, and we set that five to seven lines would be selected to be parents.

Combining methodologies that improved efficiency produced greater improvements than any single modification. Overall pipelines and modified schemes, genetic gain per cycle increased by 34.9%, genetic gain per year by 70%, gain per dollar by 35.2%, and the cost of one unit of genetic gain decreased by 24.2% (**Table 9**). The DH plus 2–2–1 tester scheme with increased selection intensity at Stages 1 and 2 improved genetic gain per cycle by 42.8%, genetic gain per year _y_ by 161.8%, gain per dollar by 43.1%, and decreased $ cost of one unit of genetic gain by 28.5% (**Table 9**). The SSD (F_6_) with the 2–2–1 tester scheme plus increased selection intensity provided similar increases for genetic gain per cycle, gain per dollar, and cost of one unit of genetic gain as the DH scheme but just one-fourth of the genetic gain per year (**Table 9**).

**Table 9:** Effect on breeding metrics of combining modified inbreeding methods, use of 2–2–1 tester scheme, and increased selection intensity from Stage 1 and Stage 2.

Inbreeding method and generation	Gain per cycle	Gain per year	Gain per dollar	Cost of one unit of gain
Pedigree, F_6_, select after Stage 3	134.9	134.9	135.4	75.4
Pedigree, F_4_, select after Stage 3	125.0	152.8	125.2	81.5
SSD, F_6_, select after Stage 3	141.4	141.4	141.7	72.2
SSD, F_4_, select after Stage 3	130.3	159.2	130.6	78.3
Doubled haploid, select after Stage 3	142.8	261.8	143.1	71.5
Average, select after Stage 3	134.9	170.0	135.2	75.8
SSD, F_6_, select after Stage 2	90.8	99.9	93.0	108.0
DH, select after Stage 2	92.8	204.1	94.9	106.2

Metric values are expressed as a percentage of values from the base (current) breeding scheme that uses the pedigree method, low selection intensity, and a 4–1–1 tester scheme. Results are averaged over the four Agricultural Research, Innovation, and Specialist Services (ARISS) Directorate breeding pipelines. The total cost of the modified schemes were constrained to equal the total cost of the base scheme.

Selection intensity could not be increased in stage 3, as these trials were quite small after higher selection intensity in Stages 1 and 2, and we were selecting five to seven parents from Stage 3 trials. We modeled selecting parents after Stage 2 with the DH plus 2–2–1 tester scheme and the SSD (F_6_) plus 2–2–1 tester scheme. This reduced the number of years per breeding cycle and the total cost, although small Stage 3 trials were not very expensive. Resources saved from not conducting Stage 3 trials were reallocated to testing more lines in Stage 1. Selection intensity in stage 2 was determined by our target number of parents (5 or 7). Across the four pipelines, the two-stage selection reduced genetic gain per cycle and gain per dollar and increased the cost of one unit of genetic gain (**Table 9**).

## Discussion

Improving the rate of gain achieved by crop breeding programs is a critical component of increasing yields in farmers’ fields in an increasingly harsh climate. While tools such as genomic selection, speed breeding, and remote sensing for high throughput phenotyping can increase gains, they can be difficult to implement in resource-constrained breeding programs (Voss-Fels et al., 2019). In addition, such technologies complement effective traditional breeding schemes. Optimizing current, traditional breeding schemes within their budget constraints is a critical first step towards increasing genetic gain and to reap the benefits of new technologies. Breeding simulation tools incorporating operational costs allow breeding programs to identify inefficiencies and predict the impact of specific changes to breeding schemes within their realities (Faux et al., 2016). The current genetic gain for grain yield within Zimbabwe’s national maize breeding program was estimated at 0.89% per year (Mukaro et al., 2024). We evaluated revised breeding schemes for Zimbabwe’s national maize breeding program and assessed their potential impact on metrics of breeding effectiveness and efficiencies. Possible inefficiencies in the current base schemes included an expensive pedigree system to generate inbred lines, a 4–1–1 tester scheme that limited the number of lines evaluated in Stage 1, and low selection intensity. The impact of SSD and DH methods to generate inbred lines, a 2–2–1 tester scheme, and increased selection intensity were investigated.

The deterministic model of Atlin and Econopouly (2022) used in the analyses is an easy to use Excel-based tool that uses the breeding equation (Equation 1), which assumes recurrent selection. Most breeders do not use strict recurrent selection as most introduce new genetics into their populations during every crossing event. Still, the equation is useful to predict the genetic gain from one cycle of selection originating from one base population where the best lines are selected and intercrossed to form a new population.

Most breeding programs are budget constrained, so we modeled changes such that every modified pipeline cost the same as the current pipeline. Any saving from a modification was reallocated to increasing the size of the Stage 1 trial, thereby increasing selection intensity at that stage. Every modification improved genetic gain. Current pipelines use a pedigree scheme to produce inbred lines. The use of DHs could significantly improve all efficiency parameters (**Table 6**). Using SSD to generate F6 inbred lines had a similar impact except on gain per year. The predicted gain associated with moving to DH technology for inbred line development could be partly explained by the very high cost of pedigree breeding. Given the high cost of the pedigree system, its use needs well-documented benefits, as it is detrimental to all the breeding metrics. Zimbabwe has a unimodal climate, and the off-season nursery is located 210 km outside of Harare. Expenses associated with managing nurseries in remote locations are currently a major cost driver of the pedigree system, and a large number of lengthy rows are required. Less inbreeding within a pedigree breeding system not only reduced the cost of inbred line development (although the reduction was relatively small) but also reduced the G_c_ as genetic variation between inbred lines was reduced, thereby reducing heritability. DH technology has been used for inbred line development in temperate maize breeding for over half a century (Andorf et al., 2019). In 2013, the first African-based DH facility was opened in Kenya (Prasanna et al., 2021). Converting to DH from pedigree line development is a simple change to operationalize and can significantly improve breeding metrics within the ARISS maize breeding program. While DH is the primary source of inbred line development at the International Maize and Wheat Improvement Center (CIMMYT) (Prasanna et al., 2022), the use by national maize breeding programs in SSA is lower. Ligeyo et al. (2024) reported that DH lines only accounts for 10% of inbred line development in the Kenyan Agricultural and Livestock Research Organization (KALRO) highland maize breeding program. Similarly, at ARISS, DH currently accounts for approximately 20% of inbred line development (Mukaro et al., 2024). Despite subsidized costs to national programs and the reduced overall cost of DH for inbred line development, the high upfront cost of DH relative to pedigree breeding, where the total cost of inbred line development is over 3 years, may reduce uptake. Reducing the cost of DH production through more efficient methods to separate haploids from diploids during production could reduce the unit cost of DH (Chaikam et al., 2017). Moving from pedigree breeding to DH would also facilitate the deployment of marker-assisted forward breeding for key diseases such as maize streak virus, reducing field phenotyping requirements and field costs. One limitation of employing DH is the number of lines per cross (50–100) delivered by the provider. At ~ USD 20 per line, the DH plan costs between $1,000 and $2,000 per cross. The SSD system can provide similar benefits for all metrics except gain per year and can be easily implemented within a program.

ARISS maize breeding pipelines currently implement a 4–1–1 tester scheme using two single-cross testers (from different heterotic groups—A and B), which allows early identification of the market-preferred three-way hybrids. The inbred line testers would serve to provide more accurate GCA estimates for the inbred lines under test, thus accounting for the four testers at the early stage. However, this requires streamlining industry testing practices and using one tester at an early stage to reduce costs. Further genetic gains were obtained by switching from the current 4–1–1 tester strategy to the 2–2–1 strategy. The current program crosses each new lines to four testers for Stage 1, thereby greatly limiting the number of new lines that could be tested. The high number of testers is because ARISS has historically used the heterotic groups: N3 (Salisbury white—N2.2.3.3) and SC (Southern cross—SC5522). ARISS collaborates with the International Maize and Wheat Improvement Center (CIMMYT), which uses the heterotic groups A and B. Thus, the use of four testers represents, in part, the alignment of requirements of two different breeding systems. The 2–2–1 tester scheme would allow twice as many lines to be evaluated in Stage 1 within the same budget, thereby significantly improving efficiency metrics (**Table 7**). Each line advanced to Stage 2 would be crossed to two other testers such that after Stage 2, each line would be evaluated with a total of four testers. Reducing the number of testers is one pathway to increasing the size of the Stage 1 trials within a fixed budget, allowing selection intensity to be increased along the stage-gate advancement process. Refining heterotic groups to inform and refine the tester strategy used by ARISS should be a priority to facilitate the implementation of a reduced number of testers (Gonhi et al., 2024).

The current pipeline employs low selection intensity as lines are advanced from Stage 1 and Stage 2. The improvements from using DHs, SSSD, or the 2–2–1 tester scheme are derived from increasing selection intensity in Stage 1. We also modeled increasing selection intensity from Stage 2 (**Tables 8**, **9**). Increasing selection intensity had the greatest impact on gain per cycle and year. Increased selection intensity has been attributed as a key driver of genetic gain in maize yields in the USA (Cooper et al., 2014) and in eastern and southern Africa (Prasanna et al., 2022). Selection intensity can be increased by expanding the size of the phenotyping network and, subsequently, using molecular breeding to increase the size of the untested layer (Cooper et al., 2014). Within the ARISS maize breeding program, the average selection intensity was >60% in the advancement of testcrosses from Stage 1 to Stage 2 and >70% in Stage 2 to Stage 3. The selection intensity at each stage is approximately half that of the CIMMYT’s maize breeding pipelines in southern Africa (Prasanna et al., 2022).

The combination of using DH lines, a 2–2–1 tester plan, and increased selection intensity had a large impact on the breeding efficiency metrics, improving gain per cycle by 42.8%, gain per year by 161.8%, gain per dollar by 43.1%, and decreasing cost of one unit of genetic gain by 28.5% (**Table 9**). These results are pipeline specific and cannot be extrapolated to other breeding programs, even if the same breeding scheme is used. Operational costs vary by pipeline, and heritability is trait and pipeline specific. However, the results of this study highlight how optimizing breeding schemes could provide a low-hanging fruit in the drive towards increasing breeding gains within crop breeding programs in the Global South. Such modifications are easy to implement and do not require additional funding. Similarly, Bernardo (2021) reports that breeding programs cannot sorely rely on phenotypic selection to enhance germplasm improvement, particularly in a cost-neutral and time-neutral breeding program.

This research shows the paramount importance of costing when comparing breeding schemes. The proposed changes in breeding schemes reiterate basic quantitative genetics principles and, as a result, appear self-evident. However, this overlooks the realities many public-sector plant breeding programs face and the “psychology and behavioral economics of plant breeders” (Cobb et al., 2019). Within the context of public sector breeding programs in the Global South, staff turnover is high (retention times are often <5 years). Within the ARISS maize breeding program, the average service of a breeder over the past two decades is 5.3 years. Implementing change requires an in-depth understanding of current breeding schemes and the historical rationale behind these schemes. Key performance indicators (KPIs) for breeders increasingly include genetic gain for key traits. KPIs help maintain focus on achieving specific short-term metrics essential for breeding progress; they risk limiting innovation and change required to achieve longer-term goals (Voss et al., 2023). Breeders, in general, are often hesitant to replace proven methods and breeding schemes in already successful breeding pipelines (Awada et al., 2018; Lenaerts et al., 2018a; Reynolds et al., 2020). Convincing breeders and leadership to implement changes within breeding schemes requires a strong value proposition and projections of the benefits of the modifications (Lenaerts et al., 2018b; Cobb et al., 2019). There are few documented examples of a cost–benefit analysis of new tools (Dreher et al., 2003; Morris et al., 2003; Biswas et al., 2024). Many breeding simulation tools do not incorporate operational costs (Sun et al., 2011; Faux et al., 2016), and they can be difficult to use, thus deterring their direct use by breeders. While operational costs can be estimated (Li et al., 2012) or a fixed cost used (Bernardo, 2021), this approach often underestimates costs, particularly for full cost recovery. The tool developed by Atlin and Econopouly (2022) provides an easy, breeder-friendly way to compare breeding schemes and their impact on key metrics within the context of their realities. More complex simulations can be used to identify further modifications to improve breeding efficiency; however, the proposed modifications based on a simple deterministic model are identified as key first steps to improvement.

## Conclusions

Simple, deterministic simulation models that utilize current genetic gains and fully costed breeding pipelines can provide a powerful tool for identifying inefficiencies and opportunities to optimize breeding schemes. Current genetic trends for grain yield within Zimbabwe’s national breeding pipeline are 0.89% per year (Mukaro et al., 2024). Our analysis suggests that implementing simple changes to the breeding scheme could double the yearly gain within the program’s current budget. While the results presented here are predictions and program specific, they demonstrate how modifications to breeding schemes can be easily modeled and highlight how improvements in breeding metrics can be achieved within fixed budget. They also illustrate that comparing breeding schemes can be easily accomplished.

## Data Availability

The raw data supporting the conclusions of this article will be made available by the authors, without undue reservation.
